# Obesity measured as percent body fat, relationship with body mass index, and percentile curves for Mexican pediatric population

**DOI:** 10.1371/journal.pone.0212792

**Published:** 2019-02-25

**Authors:** Paula Costa-Urrutia, Alejandra Vizuet-Gámez, Miryam Ramirez-Alcántara, Miguel Ángel Guillen-González, Oscar Medina-Contreras, Mariana Valdes-Moreno, Claudette Musalem-Younes, Jaqueline Solares-Tlapechco, Julio Granados, Valentina Franco-Trecu, M. Eunice Rodriguez-Arellano

**Affiliations:** 1 Laboratorio de Medicina Genómica del Hospital Regional Lic, Adolfo López Mateos, ISSSTE, Mexico City, Mexico; 2 Coordinación de Pediatría Hospital Regional Lic, Adolfo López Mateos, ISSSTE, Mexico City, Mexico; 3 Laboratorio de Investigación Clínica Hospital Regional Lic, Adolfo López Mateos, ISSSTE, Mexico City, Mexico; 4 Laboratorio de Inmunología y Proteómica, Hospital Infantil de México Federico Gómez, Mexico City, Mexico; 5 División de Inmunogenética, Departamento de Trasplantes, Instituto Nacional de Ciencias Médicas y Nutrición Salvador Zubirán, Mexico City, Mexico; 6 Departamento de Ecología y Evolución, Facultad de Ciencias, Universidad de la República, Montevideo, Uruguay; Università degli Studi di Milano, ITALY

## Abstract

In Mexico, the increase in childhood obesity is alarming. Thus, improving the precision of its diagnosis is expected to impact on disease prevention. We estimated obesity prevalence by bioimpedance–based percent body fat (%BF) and body mass index (BMI) in 1061 girls and 1121 boys, from 3 to 17 years old. Multiple regressions and area under receiver operating curves (AUC) were used to determine the predictive value of BMI on %BF and percentile curves were constructed. Overall obesity prevalence estimated by %BF was 43.7%, and by BMI it was 20.1%; it means that the diagnosis by BMI underestimated around 50% of children diagnosed with obesity by %BF (≥30% for girls, ≥25% for boys). The fat mass excess is further underestimated in boys than in girls when using the standard BMI classification. The relationship between %BF and BMI was strong in school children and adolescents (all cases R^2^>0.70), but not in preschool children (girls R^2^ = 0.57, boys R^2^ = 0.23). AUCs showed greater discriminative power of BMI to detect %BF obesity in school children and adolescents (all cases AUC≥0.90) than in preschool children (girls AUC = 0.86; boys AUC = 0.70). Growth percentile charts showed that girls aged 9–17 years and boys aged 8–17 years presented fat excess from the 50^th^ percentile and above. We suggested to change the BMI cut-off for them, considering values at the 75^th^ percentile as overweight, and values at the 85^th^ percentile as obesity, as previously recommended for Mexican children. Improving obesity diagnosis will allow greater efficiency when searching for comorbidities in clinical practice.

## Introduction

Childhood obesity is the result of an increase in body weight greater than expected with respect to height, which generates excessive body fat. This excess of body fat in children and adolescents can lead to a variety of clinical conditions such as type 2 diabetes (T2D) [1], cardiovascular disease [2], sleep apnea and non-alcoholic fatty liver, which contribute to an increase in morbidity and/or premature mortality [3].

The obesity epidemic, currently affecting pediatric population, has become a major global health challenge [4]. Multiple environmental factors contribute to the globally high prevalence: increase in birth weight, fast food, lack of physical activity and maternal smoking in the first year of life [5]. In Mexico, the increase in prevalence of overweight and obesity for children and adolescents is particularly evident in the last two decades. In school children it increased from 25% in 1999 to 32.2% in 2012, and in adolescents from 11.1% in 1999 to 36.4% in 2012 [6].

Body mass index (BMI) is the most widely used proxy to assess overweight and obesity in children and adults. Although BMI is simple to measure and low cost, it does not distinguish between fat and lean mass tissue and, thus, it may lead to misclassification [7]. Besides, it may not appropriately reflect changes in fat mass during childhood obesity treatment [8]. In addition, BMI was recently found not to be the best anthropometric predictor for Metabolic Syndrome neither for cardiovascular disease in European adult population [9]. The BMI obesity misclassification has been reported in Mexican and Asian populations, which presents lower mean heights than European population. Mexicans or Asians have higher body fat values for a given BMI when they are compared with Europeans [10,11]. Hence, directly measuring body fat becomes relevant to determine obesity. In this regard, research and application of bioimpedance for body composition measurements have been growing fast in clinical practice, as the technique is non-invasive and low cost. Furthermore, bioimpedance analyses have gained precision, and is regarded as a suitable technique to obtain estimations of lean mass or fat mass [12,13]. For children, there are no accepted international standard cut-offs for excess of body fat, commonly reported as percent body fat (%BF). However, several studies have reported that 30%BF for girls and 25%BF for boys are associated with low high lipoprotein density, high triglycerides, high total cholesterol, blood pressure and cardiovascular disease [14–16].

Given the widespread use of BMI cut-off values for classifying overweight and obesity, it is important to assess the adiposity cut-off values predicted from those BMI cut-off values in children of diverse ethnicities. In this study we a) estimated the prevalence of obesity using %BF and compared it with the World Health Organization (WHO) BMI cut-off for overweight and obesity, b) evaluated the relationship between %BF and BMI, and c) estimated percentile curves of %BF and BMI.

## Materials and methods

We used anthropometric data of children and adolescents between 3 and 17 years old from COIPIS cohort (Childhood obesity cohort of the Healthy childhood project) of the Genomic Medicine Laboratory at Regional Hospital Lic. Adolfo López Mateos of the Institute of Security and Social Services of State workers (ISSSTE). COIPIS cohort started in 2012 in response to the need for establishing a prospective childhood obesity epidemiologic study. The sample consisted of 1061 girls and 1121 boys, from 3 to 17 years old. Participants were invited among ISSSTE right-holder teachers who were also members of the union, and patients treated at ISSSTE clinics distributed in Mexico City. Participants were examined every 6 months, since they were recruited until they turned 17 years old.

Weight, height and %BF were measured through InBody J10 equipment (Gangnam-gu, Seoul 135–854 KOREA). Children were measured after a 10-hour fast, without consuming water in the morning before being measured, and they were barefoot and wearing light clothes. Accuracy of the stadiometer integrated to InBody J10 was ±0.1 cm and ±0.01 kg for height and weight respectively. Electrical bioimpedance was used to estimate %BF as implemented in InBody J10 tetrapolar equipment of three frequencies (5, 50, and 250 kHz) and anthropometry [17].

BMI (kg/m^2^) was calculated as body weight (kg) divided by square height (m^2^). Classification in overweight and obesity was done following WHO cut-off of BMI in relation to sex and age [18,19]. As there is no standard statement of %BF cut-offs to define overweight, we estimated means and standard deviation of %BF of children classified as overweight and obesity by BMI.

We chose a health-related definition of %BF obesity. We estimated prevalence of ≥30%BF for girls and ≥25%BF for boys, as these cut-offs have been significantly associated with cardiovascular risk factors in children and adolescents [14]. In addition, it is widely used in studies on the relationship between BMI and %BF in children and adolescents [20,21].

This project was approved by the Ethics Committee of Regional Hospital Lic. Adolfo López Mateos, ISSSTE, México (Number 245.2012). All parents of the participants provided written informed consent prior to their inclusion in the study.

### Statistical analysis

We stratified our data in three age groups corresponding to preschool (3 to 5 years old), school (6 to 11 years old) children, and adolescents (12 to 17 years old). BMI overweight and obesity, and %BF obesity prevalence were estimated in the total sample, according to sex and age group. Means and standard deviation were used to describe measurements by sex and age group. Mann-Whitney test was used for pairwise group comparisons using XLSTAT (Data Analysis and Statistical Solution for Microsoft Excel. Addinsoft, Paris, France 2017)

The relationship between BMI and %BF was assessed through a multiple regression, where %BF was used as a response variable and BMI and age as predictive variables, using XLSTAT. The analysis was done by sex and age group. Variance inflation factor (VIF) was calculated to know the co-linearity between predictors (BMI and age), with high co-linearity (VIF>5) implying poor estimates. Q-Q plots were carried out for inspection of %BF normal distribution. All linear models were subjected to the customary residual analysis.

The discriminative power of BMI to detect obesity by %BF was tested by calculation of the area under the receiver operating characteristic (ROC) curves (AUC) using XLSTAT. ROC curves represent the proportion of true positive cases (sensitivity) as a function of the proportion of false positives cases (corresponding to 1-specificity) [22]. The AUC ranges from 0 to 1, where 1 is a perfect score and 0.5 indicates results not better than chance. It reflects whether a randomly selected participant with obesity has a greater BMI value than that of a randomly selected participant without obesity. ROC curves were assessed by sex and age group.

Percentile curves for %BF and BMI were constructed using the lambda mu sigma (LMS) method [23] using R Development Core Team (2013). The M and S curves correspond to the median and coefficient of variation of %BF and BMI at a given sex and age, whereas the L curve allows for substantial age-dependent skew in the distribution of %BF and BMI. The assumption underlying the LMS method is that data at each age is normally distributed after Box-Cox power transformation. %BF and BMI were estimated from those values using the following equation: M*(1-LSz)^1/L^, where L, M and S are values of the fitted curves at each age, and z indicates the z-score for the required centile.

## Results

This study included 2,182 children aged between 3 and 17 years (girls = 1,061, boys = 1,121). Descriptive results as mean, standard deviation, coefficient of variation and Box-Cox transformation parameter (L) by sex and age are shown in S1 Table.

Overall %BF obesity prevalence was 43.7% and BMI overweight-obesity prevalence was 42.7%. The highest %BF obesity prevalence and BMI overweight-obesity prevalence for girls were found in adolescents, and for boys they were found in school children, since more than half of these age groups presented obesity or overweight-obesity. For preschool and school children there were no significant differences in BMI between girls and boys classified as overweight and obesity. Meanwhile, for those categories, adolescent girls showed BMI values significantly higher than boys. Boys presented significantly higher %BF than girls, except for girls classified as overweight. Preschool, school children and adolescents, both girls and boys, classified as overweight by BMI showed mean values over 30%BF, and children classified with obesity by BMI showed mean values of over 40%BF, except for preschool girls classified with obesity who showed 36%BF (Table 1).

**Table 1:** Means, standard deviation (SD) and prevalence (in percentage) for body mass index (BMI) and percent body fat (%BF) for children classified with overweight (OW) and obesity (OB) by BMI, by age group and sex.

		Preschool children			School children			Adolescents	
Mean (SD)	Girls	Boys	p	Girls	Boys	p	Girls	Boys	p
BMI-OW	19.0 (2.2)	19.3 (0.8)	0.6	25.0 (2.5)	24.4 (3.3)	0.6	32.5 (5.1)	30.1 (3.2)	0.0007
%BF-OW	32.0 (1.8)	38.0 (4.89)	0.02	38.4 (4.6)	41.5 (5.1)	0.0001	44.6 (1.3)	43.0 (5.6)	0.006
BMI-OB	19.2 (0.9)	19.7 (0.5)	0.08	27.2 (2.7)	26.0 (3.4)	0.3	35.0 (3.9)	32.6 (4.5)	0.03
%BF-OB	36.3(1.7)	43.2 (0.5)	0.008	40.6 (4.2)	49.6 (5.1)	0.0001	47.0 (1.2)	50.6 (5.2)	0.001
**Prevalence**									
BMI-OW	16.4 (n = 19)	17.9 (n = 23)		25.9 (n = 132)	22.8 (n = 133)		28.9 (n = 126)	23.4 (n = 96)	
%BF-OW	9.7 (n = 11)	7.8 (n = 10)		10.5(n = 53)	10 (n = 59)		11.6(n = 50)	9.1 (n = 39)	
BMI-OB	10.3 (n = 12)	5.5 (n = 7)		22.4 (n = 114)	31.2 (n = 182)		22.1 (n = 96)	28.8 (n = 118)	
%BF-OB	7.1 (n = 8)	0.8 (n = 2)		5.4 (n = 27)	2.6 (n = 15		6.0 (n = 26)	5.3 (n = 23)	
Health %BF	21.6 (n = 25)	28.9 (n = 37)		41.8 (n = 213)	53.7 (n = 313)		65.5 (n = 285)	49.3 (n = 202)	

Preschool children (3–5 years old), School children (6–11 years old), Adolescents (12–17 years old), Health %BF = Health related cut off value for %BF (%BF ≥ 30% for girls and ≥ 25% for boys classified as obesity), n = number of individuals, p = p-value.

### Relationship between %BF and BMI

BMI was a positive predictor of %BF in girls and boys of the three age groups. The relationship between %BF and BMI is greater in school children and adolescents, than in preschool children (school children and adolescents, for girls and boys R^2^>0.70; preschool girls R^2^ = 0.57, boys R^2^ = 0.23). The linear relationship between %BF and BMI was greater in girls (R^2^ = 0.77) than in boys (R^2^ = 0.72). Co-linearity did not influence the analysis (VIF<5 in all cases) (Table 2).

**Table 2:** Linear model results by sex and age group.

Predictor	β	P	VIF	R^2^	β	P	VIF	R^2^
**Girls**					**Boys**			
Constant	-2.7				19.1	<0.001		
BMI	1.8	<0.001			1.8	<0.001		
Age	-0.4	<0.001	1.5	77.2%	-2.5	<0.001	1.1	72.7%
**Preschool children**								
Constant	-17	<0.001			-7.8	0.19		
BMI	2.8	<0.001			2.2	<0.001	1	
Age	-1.4	0.02	1.1	57.1%	-1.2	0.09	1	23.0%
**School children**								
Constant	-11	<0.001			-11.8	<0.001		
BMI	2.4	<0.001			2.2	<0.001	1.2	
Age	-0.7	<0.001	1.3	80.2%	-0.3	0.01	1.2	77.5%
**Adolescents**								
Constant	4.9	0.01			19.1	<0.001		
BMI	1.5	<0.001			1.8	<0.001	1.1	
Age	-0.5	<0.001	1	80.3%	-2.5	<0.001	1.1	72.7%

Percent body fat (%BF) used as response variable, body mass index (BMI), and age (continuous variable) as predictor variables. Estimates (β), p-value (p), variance inflation factor (VIF), proportion of explained variance (R^2^) for girls, boys and age group preschool children (3–5 years old), school children (6–11 years old) and adolescents (12–17 years old).

### ROC curves

**Fig 1:**
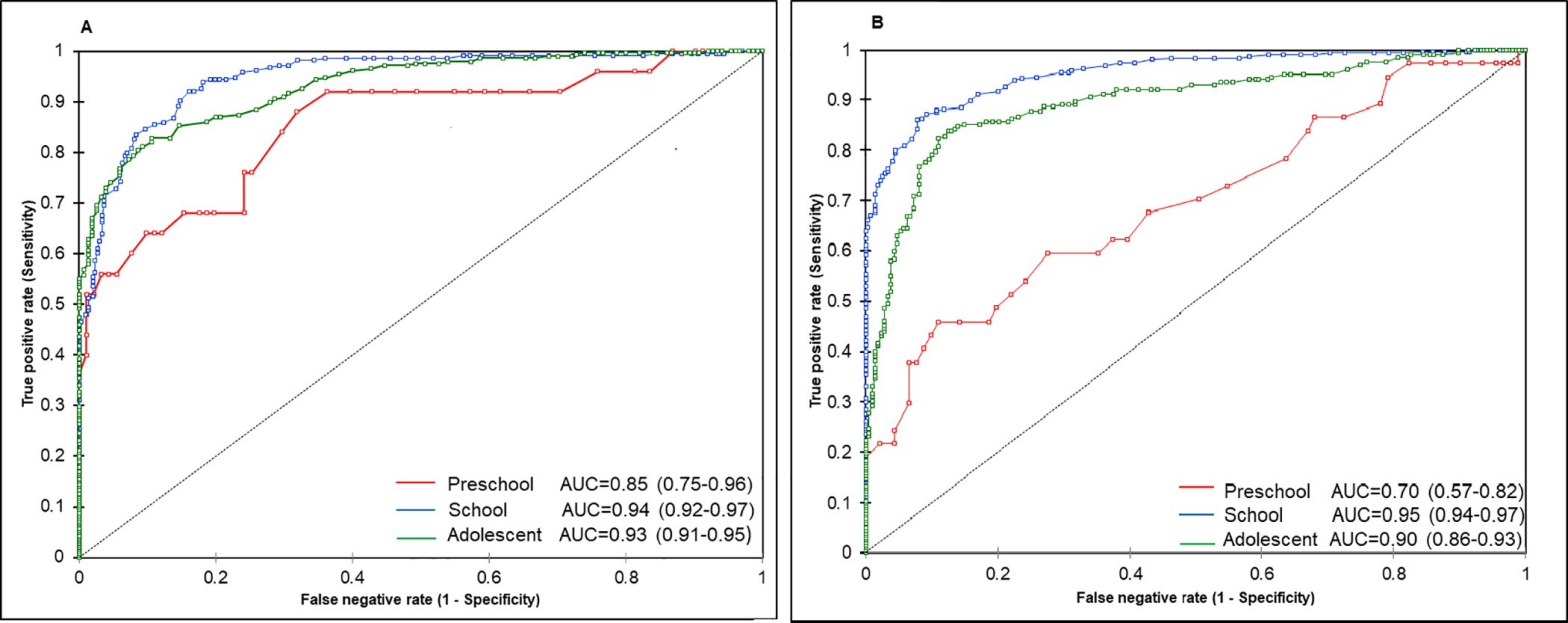
Power of body mass index (BMI) to detect obesity by percent body fat (%BF). Area under the curve (AUC) from Receiver operating characteristic curves (ROC) for BMI predicting obesity by %BF for girls (A) and boys (B) (%BF ≥ 30% for girls and ≥ 25% for boys) by age group: preschool children (3–5 years old), school children (6–11 years old) and adolescents (12–17 years old), respectively.

AUC values ranged from 0.85 to 0.95 in children of all age groups; this indicated that the ability of BMI to predict obesity through %BF was greater than chance. AUC showed BMI performed better in school children and adolescents (school children and adolescent, for girls and boys AUC≥0.90) than in preschool children (girls AUC = 0.86, Fig 1A; boys AUC = 0.70, Fig 1B). Sensitivity was greater for girls than for boys (preschool girls = 0.88, school girls = 0.92; preschool boys = 0.45, school boys = 0.86), except in adolescents, which presented no differences (girls = 0.83, boys = 0.84). Meanwhile, specificity was greater for boys than for girls (preschool girls = 0.68, school girls = 0.83, preschool boys = 0.89, school boys = 0.92), also except in adolescents, for which a slight difference was found (girls = 0.89, boys = 0.87).

### Smoothed percentile curves by LMS method

**Fig 2:**
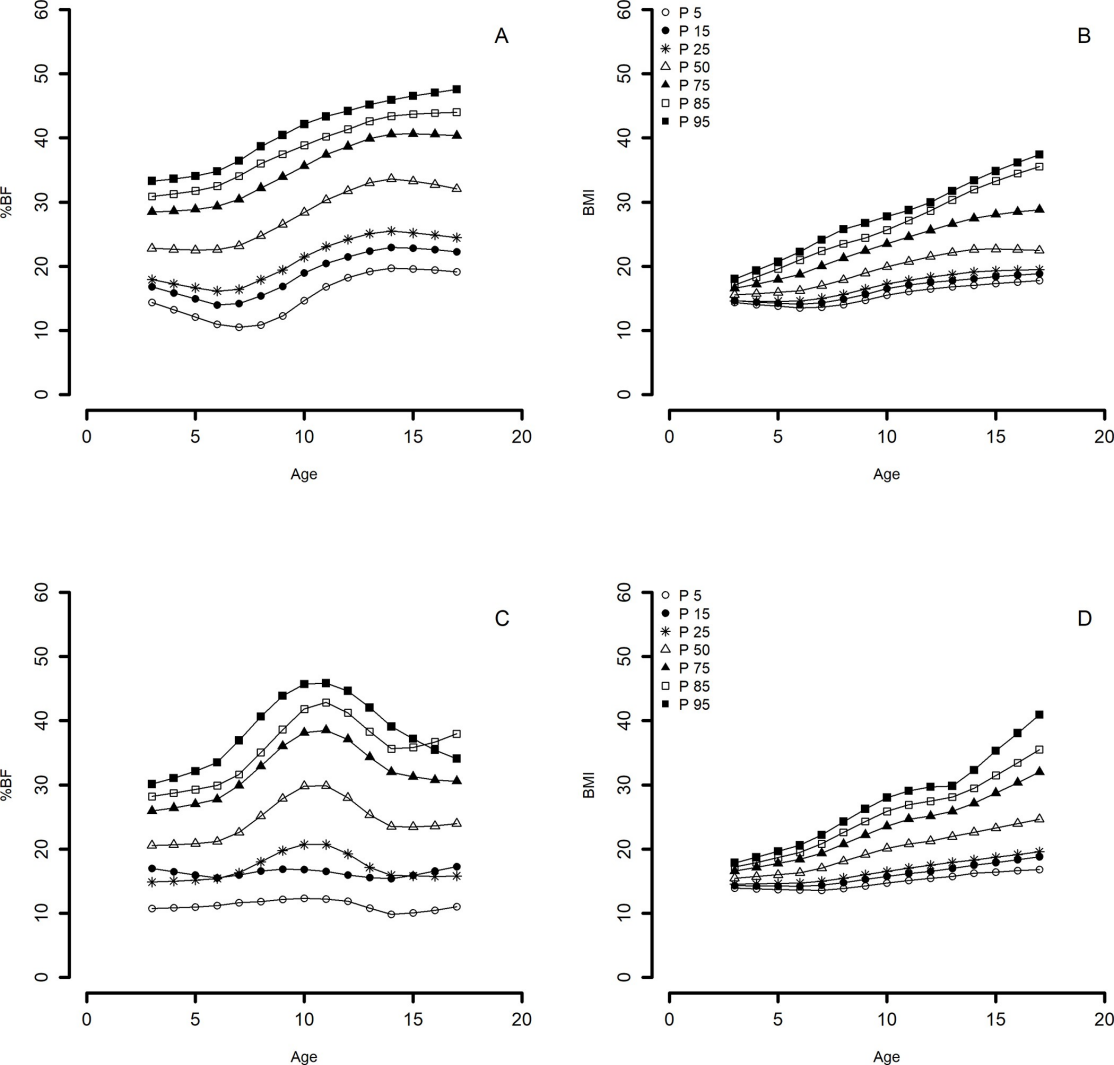
Percentile (P) curves. Percent body fat (%BF) and body mass index (BMI) for girls (A) and (B) and for boys (C) and (D) by LMS method.

Values for %BF and BMI percentiles (5th, 15th, 25th, 50th, 75th, 85th, 95th) of LMS curves, and tables with reference values of WHO-BMI according to age and sex are shown in Fig 2 (Fig 2A, Fig 2B for girls and Fig 2C, Fig 2D for boys) and Table 3. Girls aged 3–8 years showed 30%BF in the 75th percentile, whereas girls aged 9–17 years were in the 50th percentile (Table 3). Girls showed a growing tendency to increase %BF, with a more pronounced growth after 9 years old (Fig 2A). Boys aged 3–6 years, showed %BF closer to 25% in 75th percentile, whereas those aged 7–13 years were in the 50th percentile. Boys aged 14–16 years were near the 75th percentile and those aged 17 years were near the 50th percentile. Boys showed a constant tendency in %BF between 3 and 6 years old, with a pronounced growth curve between 7 and 12 years old, decreasing until 15 years old and increasing again at 17 years old (Fig 2C). For BMI, both girls and boys showed a gradual increase, but more pronounced from the 50th percentile and above (Fig 2B, Fig 2D). Girls and boys in this study had higher BMI percentiles as compared with WHO reference growth chart across the whole range of age analysed (Table 3).

**Table 3:** Girls and boys percentile (P) values of percent body fat (%BF), World Health Organization (WHO)-body mass index (BMI) references, and BMI estimated by lambda mu sigma (LMS) method by age (3–17 years old).

		Girls							Boys						
Age	Variables	P5	P15	P25	P50	P75	P85	P95	P5	P15	P25	P50	P75	P85	P95
3	WHO-BMI	13.4	14.4	15.5	15.4	16.3	16.8	17.8	13.7	14.4	14.8	15.6	16.4	16.9	17.8
	BMI	14.5	14.7	14.8	15.9	17.3	18.0	19.1	13.9	14.4	14.7	15.7	16.8	17.2	17.7
	%BF	14.8	16.9	18.6	24.0	30.0	32.5	35.0	14.2	17.4	18.5	21.2	28.6	31.9	35.9
4	WHO-BMI	13.2	13.9	14.4	15.3	16.3	16.8	17.9	13.4	14.1	14.5	15.3	16.2	16.7	17.6
	BMI	14.1	14.5	14.6	15.7	16.9	17.4	18.0	13.9	14.3	14.5	15.4	16.9	18.0	19.1
	%BF	13.2	16.3	16.8	21.5	27.0	29.5	31.5	8.8	12.6	16.8	20.6	29.1	34.8	42.5
5	WHO-BMI	13.1	13.8	14.3	15.2	16.3	16.9	18.1	13.4	14.0	14.4	15.3	16.2	16.7	17.7
	BMI	13.4	13.9	14.1	15.2	17.1	19.5	20.9	13.5	14.1	14.4	15.9	17.8	18.6	19.8
	%BF	10.5	13.4	15.7	22.3	29.0	31.7	34.3	12.48	15.54	17.76	21.30	25.90	29.90	38.31
6	WHO-BMI	13.1	13.8	14.3	15.3	16.4	17.1	18.4	13.4	14.0	14.5	15.3	16.3	16.8	17.9
	BMI	13.7	14.3	14.7	16.2	18.4	20.4	21.2	13.3	13.9	14.5	16.2	18.0	18.9	19.6
	%BF	12.3	14.7	16.5	21.8	27.4	30.2	32.3	12.1	14.9	16.5	20.9	25.4	29.0	37.3
7	WHO-BMI	13.1	13.9	14.4	15.4	16.6	17.4	18.9	13.5	14.2	14.6	15.5	16.5	17.1	18.3
	BMI	13.2	13.9	14.4	16.5	20.1	23.7	25.2	14.2	14.7	15.6	16.9	19.1	20.3	21.3
	%BF	9.74	13.1	16.1	23.8	31.8	35.0	37.9	12.3	15.8	17.2	21.40	30.3	33.20	39.2
8	WHO-BMI	13.3	14.1	14.6	15.7	17.0	17.9	19.5	13.7	14.4	14.8	15.8	16.9	17.5	18.9
	BMI	13.4	14.0	16.0	18.4	22.1	23.8	25.7	12.9	13.9	14.7	17.2	20.7	22.5	24.4
	%BF	9.16	12.9	15.7	23.7	31.1	35.5	36.9	15.7	16.6	18.3	24.2	35.4	40.8	52.0
9	WHO-BMI	13.6	14.4	15.0	16.1	17.6	18.5	20.3	13.9	14.6	15.1	16.1	17.3	18.8	19.5
	BMI	15.2	15.9	16.0	18.4	22.1	23.8	28.2	14.1	15.3	16.1	19.7	23.8	25.7	27.9
	%BF	11.5	18.4	22.6	31.8	39.2	41.8	44.3	13.3	17.3	20.9	29.1	37.1	41.8	45.9
10	WHO-BMI	14	14.8	15.4	16.7	18.2	19.2	21.2	14.2	14.9	15.4	16.5	17.8	18.6	20.3
	BMI	15.1	16.5	17.5	20.0	23.3	25.0	26.2	15.7	16.6	17.8	20.8	23.9	25.8	27.4
	%BF	14.8	18.4	21.4	28.9	36.2	39.1	41.7	10.3	19.3	25.2	32.9	37.8	37.8	37.8
11	WHO-BMI	14.4	15.3	16.0	17.3	19.0	20.0	22.2	14.5	15.3	15.8	17.0	18.4	19.3	21.2
	BMI	16.8	17.8	18.5	21.5	24.8	27.5	28.4	14.9	15.9	16.9	20.3	24.7	26.9	29.5
	%BF	18.2	20.9	23.1	30.2	37.0	39.1	43.4	11.8	16.3	20.4	31.4	38.4	41.7	44.8
12	WHO-BMI	15	15.9	16.6	18.1	19.9	21.0	23.4	14.9	15.8	16.3	17.6	19.1	20.1	22.2
	BMI	16.5	17.5	18.1	21.2	26.0	29.5	31.8	14.7	16.2	17.1	21.4	25.4	28.5	30.9
	%BF	19	22.4	25.0	32.1	39.2	42.5	44.8	14.3	16.3	19.9	29.0	40.61	45.2	51.5
13	WHO-BMI	15.5	16.6	17.3	18.9	20.8	22.0	24.5	15.4	16.4	17	18.3	19.9	21	23.2
	BMI	16.2	17.5	18.5	21.9	25.5	28.2	29.1	16.8	17.7	18.7	21.7	25.4	27.4	29.4
	%BF	17.5	21.7	24.8	32.6	39.2	42.0	44.1	12.8	15.2	17.9	25.9	35.0	39.7	47.8
14	WHO-BMI	16.1	17.2	18	19.6	21.6	22.9	25.6	16.3	17.3	18	19.5	21.3	22.4	24.8
	BMI	17.8	18.3	20.1	23.7	28.5	32.2	33.7	15.3	16.4	17.4	20.6	24.5	26.4	28.4
	%BF	20.8	23.0	30.3	35.9	43.9	44.4	47.1	13.1	14.8	16.0	19.4	28.1	34.8	44.4
15	WHO-BMI	16.5	17.7	18.5	20.3	22.4	23.7	26.4	16.6	17.6	18.3	19.8	21.7	22.9	25.3
	BMI	17	18.3	19.1	22.7	28.1	34.4	36.2	17.5	18.7	20.0	23.6	28.0	30.0	31.7
	%BF	20.8	24.4	26.7	33.8	40.7	43.6	46.1	12.6	15.8	17.8	22.2	34.2	39.4	48.3
16	WHO-BMI	16.8	18.1	18.9	20.7	22.9	24.3	27	17.1	18.2	19	20.6	22.5	23.7	27.3
	BMI	18	19.0	20.1	23.7	29.4	35.2	36.8	15.9	17.4	18.5	23.4	30.4	33.9	39.1
	%BF	19.5	23.3	25.3	33.5	41.4	44.7	47.8	14.5	15.8	17.0	23.4	32.2	36.1	39.9
17	WHO-BMI	17	18.3	19.2	21.1	23.3	24.7	27.4	17.6	18.7	19.5	21.2	23.2	24.5	27.0
	BMI	17.5	18.6	19.0	21.6	26.6	34.4	36.4	16.9	19.1	19.9	24.8	32.1	35.6	40.7
	%BF	18.4	21.0	23.5	30.9	39.4	43.3	47.1	15.9	18.1	19.9	26.2	38.2	58.9	61.1

## Discussion

In this study, we estimated the obesity prevalence through %BF and BMI, evaluated the relationship between %BF and BMI, and constructed %BF and BMI percentile curves in 2182 Mexican children from 3 to 17 years old.

We found differences in %BF between girls and boys in overweight and obesity categories by BMI. Meanwhile, in preschool and school children there were no significant differences between girls and boys in BMI, and boys showed significantly higher %BF. This tendency was reversed only in girls with overweight and showing higher %BF than boys. Differences between BMI and %BF have been reported [21,24,25]: e.g. Asian girls show higher %BF even when boys showed higher BMI [25]. Our study suggests that excess of fat is further underestimated in boys than in girls under the standard classification by BMI.

Our results showed that around half of the school children and adolescents present obesity, as estimated by health-related %BF (girls = 48.1%, boys = 53.6%). Such prevalence is higher than reported in most studies in other countries using the same health-related %BF cut-off as used in this study (China: n = 2,191,girls = 34.0%, boys = 24.4% Li et al. 2015; Canadian adolescents of Asian ancestry: n = 944, girls = 31.3%, boys = 28.1%[21]; Caribbean adolescents of African ancestry: n = 3,479, girls = 43.6% boys = 12.2% [26]; US adolescents of Caucasian, Mexican and African ancestry: n = 12,279,girls = 20.0%,boys = 19.0% [15]. This is in agreement with the alarming increase in obesity in Mexico, which has been reported for over a decade [27].

Obesity prevalence estimation through %BF was near the overweight-obesity prevalence estimation through BMI for all age groups (Table 1). For instance, for the overall sample %BF obesity prevalence resulted in 43.7%, while BMI overweight prevalence was 22.6% and obesity was 20.1% (BMI overweight/obesity = 42.7%). It implies that at least half the number of individuals showing excess of fat estimated through %BF was also classified as showing obesity when estimated through BMI. This pattern was shown for school children and adolescents. Most girls and boys classified as overweight by BMI had more than 30%BF and 25%BF respectively; thus, it means that BMI underestimated near 50% of children classified with obesity by %BF. A similar underestimation of BMI to identify individuals under risk of obesity has been reported in Canadian population, where BMI classified less than half of those with obesity as defined by %BF [21].

The strong relationship between %BF and BMI observed herein has been reported in several studies [25,28]. In our Mexican children, BMI also resulted in a strong predictor through %BF in school children and adolescents, but not in preschool population for which the explained variance was lower (57% for girls and 23% for boys).

Also, ROC curves performed better for school children and adolescents than for preschool children (Fig 1A, Fig 1B). Sensitivity of BMI as a predictor of %BF obesity in school children was higher in girls (0.92) than in boys (0.86), which means that 8% of girls and 14% of boys were not classified as presenting obesity through BMI when they actually presented an excess of fat. For adolescents, sensitivity was similar between sexes (girls = 0.83, boys = 0.84); around 17% of girls and boys with excess of adiposity were not classified as presenting obesity. The sensitivity reported in this study is higher than that of an extensive meta-analysis encompassing 37 studies (Sensitivity: girls = 0.73, boys = 0.67) [29], suggesting that BMI has high discriminative power to detect %BF obesity in Mexican school children and adolescents. However, caution is needed since 8–17% of school children and adolescents may show healthy BMI values, though they may have excess of adiposity. For preschool children, ROC curves showed that BMI did not classify correctly a high proportion of children (AUC: girls = 0.85, boys = 0.70, Fig 1A, Fig 1B). In particular, sensitivity for boys was less than that for girls (Sensitivity: girls = 0.88, boys = 0.45), leaving 55% of boys with fat excess out of the classification of obesity.

Growing tendency of increasing %BF showed by our smooth percentile curves (Fig 2A) agreed with the expected standard patterns of growth in girls; females deposit fat as a natural part of the ontogeny of their sexual and reproductive physiology [30]. Our curves also agreed with the %BF LMS curves for girls aged 6–12 years from a recent study in Mexico City [31]. In our study, pubescent and adolescent (9–17 years old) girls reached 30%BF in the 50th percentile (Fig 2A, Table 3) and showed significantly higher %BF than boys in the overweight category (Table 1). A similar pattern was reported for US girls between 8 and 19 years old in the 50th percentile, who present values of 30%BF along the range of age analysed [32]. This pattern is not present in UK girls, from 16 years old or older, who present 30% BM from the 85th percentile [12].

The pattern of %BF observed in our curves for boys, i.e. an increase between 7 and 13 years old and a decrease between 14 and 16 years old, is also typical growth for the age (Fig 2C). At puberty, sex hormones induce a pronounced sexual dimorphism: males gain proportionately more muscle and lean tissue than fat [30]. Our results also agreed with the growing pattern showed for boys aged 6–12 years in Mexico City [31]. However, we observed an increase in %BF for adolescents aged 17 years (Fig 2C, Table 3). Even when this increase in %BF is not the expected standard growth pattern, it could be explained by the high overweight-obesity prevalence in Mexican adults (70%), which is even higher for men than for women [33].

Our percentile tables for boys aged 3–6 years showed values closer to 25%BF in the 75th percentile. In puberty (7–13 years old) boys reach 25%BF in the 50th percentile, whereas in late adolescence (14–16 years old) they do so in the 75th percentile (Table 3). This pattern is also similar to that reported for American boys, who present 25%BF in the 50th percentile between 8 and 13 years old, and around the 70th percentile from 14 years old onwards [32]. Again, this pattern is not present in UK boys, from 10 to 12 years old, who present 25%BM in the 91th percentile [12].

According to two Mexican extensive studies, one including children aged 10–18 years (n = 18528) [27] and the other including children aged 6–12 years (n = 3514) [31], and our study (children aged 3–17 years), Mexican have higher BMI percentiles as compared with WHO reference growth chart. Along with the mean %BF (>30% for overweight and ≥40% for obesity, except for preschool girls), it suggests that the BMI cut-off 85th and 95th percentiles as equivalents of overweight and obesity from reference population should no longer be used in the Mexican population.

The European Society of Endocrinology and the Pediatric Endocrine Society recommends, for prevention purposes, the evaluation of obesity comorbidities in children and adolescents with BMI ≥85th percentile [34]. In 2015, The Prevention, Diagnosis and Treatment of childhood obesity Mexican Experts Committee, suggested to adequate the BMI cut-offs due to the high risk the population presented of developing components of metabolic syndrome. They suggested to consider values higher than the 75th percentile as overweight, and those located in the 90th percentile as obesity [35]. Taking into account both suggestions and our results, for prevention purposes, we strongly recommend to consider the 75th percentile as overweight and the 85th percentile as obesity, at least until no overweight and obesity sex-age-dependent %BF cut-off is established for Mexican children.

Improving our diagnosis of obesity will allow a more efficient search for comorbidities associated with obesity, such as cardiovascular risk through lipid profile, T2D through glucose, maternal clinical history as gestational diabetes, obesity and T2D, in order to prevent further health complications [34,35].

A major limitation of the current study is the %BF cut-offs used to define obesity, since they are not age-dependent and no overweight cut-off is available. Ideally, overweight and obesity should be defined as different %BF cut-offs not only by sex, but also by age. The unique cut-off across the whole range of age may overestimate or underestimate obesity prevalence according to age [36]. Currently, there is not an accepted statement of %BF cut-offs to define overweight or obesity by age. Therefore, further studies should be conducted to identify them by using metabolic health-related criteria [25]. Although %BF measured by bioimpedance is more representative of the body fatness than BMI, it is less accurate than other methods such as DXA. However, the relatively inexpensive, portable, non-invasive and simple to use features of the bioimpedance method [12], makes it an excellent tool for clinical and public health.

Thus, it is needed to gain precision on overweigh and obesity classification, adding the cardiometabolic risk factor in large-scale studies. Finally it is importantly to bear in mind that our body fat curves are representative to Mestizo population, we do not recommend them to be used for Amerindian ethnic groups.

In conclusion, we validated the BMI as a predictor of %BF in school children and adolescents (6–17 years old) due to the high linear relationship, high AUC and sensitivity values that they presented. However, we observed a different situation in children from 3 to 5 years old, in which we strongly recommend using %BF as a predictor of obesity. With the aim of gaining precision in the classification of obesity, and for preventive purposes, we suggest to change the BMI cut-off for obesity.

## Supporting information

S1 Table Percentage body fat and body mass index, number (N), median (M), standard deviation (SD), coefficient of variation (CV) and box cox transformation parameter (L) for girls and boys and age. (DOCX)
