# Supplementary material for: Obesity measured as percent body fat, relationship with body mass index, and percentile curves for Mexican pediatric population
Source: PLoS One. 2019 Feb 25;14(2):e0212792. doi: 10.1371/journal.pone.0212792 (PMC6388924; doi:10.1371/journal.pone.0212792)
Supplement: S1 Table — (DOCX) [file pone.0212792.s001.docx]

S1 Table. Percentage Body Fat and Body Mass Index, Number (N), Median (M), Standard deviation (SD), coefficient of variation (CV) and Box Cox transformation parameter (L) for girls and boys and age.

|  |  | **Body Fat Percentage** | | |  |  | **Body mass index** | | |  |
| --- | --- | --- | --- | --- | --- | --- | --- | --- | --- | --- |
| **Age** | **N** | **M** | **SD** | **CV** | **L** |  | **M** | **SD** | **CV** | **L** |
| **Girls** |  |  |  |  |  |  |  |  |  |  |
| 3 | 24 | 24.500 | 6.693 | 0.260 | 0.56 |  | 16.100 | 1.288 | 0.079 | -5.000 |
| 4 | 43 | 22.000 | 6.192 | 0.267 | 0.66 |  | 15.800 | 1.235 | 0.078 | -1.000 |
| 5 | 49 | 22.600 | 8.274 | 0.336 | 0.96 |  | 16.200 | 2.529 | 0.150 | -5.000 |
| 6 | 77 | 22.200 | 7.361 | 0.306 | 0.82 |  | 16.200 | 2.148 | 0.129 | -2.330 |
| 7 | 95 | 24.200 | 9.928 | 0.376 | 1.02 |  | 16.600 | 3.237 | 0.185 | -2.040 |
| 8 | 74 | 24.000 | 9.811 | 0.366 | 1.15 |  | 16.650 | 3.413 | 0.191 | -3.000 |
| 9 | 81 | 32.100 | 9.202 | 0.289 | 1.85 |  | 19.200 | 3.608 | 0.183 | -1.680 |
| 10 | 95 | 29.300 | 9.120 | 0.292 | 1.24 |  | 20.000 | 3.579 | 0.176 | -1.100 |
| 11 | 88 | 30.450 | 8.238 | 0.267 | 0.78 |  | 21.250 | 3.496 | 0.168 | -0.510 |
| 12 | 99 | 32.500 | 8.414 | 0.258 | 1.06 |  | 21.400 | 4.442 | 0.200 | -1.310 |
| 13 | 81 | 32.900 | 8.534 | 0.252 | 1.58 |  | 21.900 | 4.336 | 0.189 | 0.170 |
| 14 | 88 | 34.800 | 8.886 | 0.245 | 1.73 |  | 23.700 | 5.012 | 0.203 | -0.660 |
| 15 | 64 | 34.100 | 8.139 | 0.236 | 1.15 |  | 22.750 | 5.404 | 0.226 | -1.480 |
| 16 | 69 | 33.800 | 9.498 | 0.269 | 0.94 |  | 23.700 | 6.480 | 0.258 | -1.250 |
| 17 | 34 | 31.350 | 9.755 | 0.290 | 0.45 |  | 22.700 | 5.787 | 0.235 | -3.110 |
| **Boys** |  |  |  |  |  |  |  |  |  |  |
| 3 | 33 | 21.200 | 6.881 | 0.293 | 0.900 |  | 15.800 | 1.201 | 0.076 | 0.630 |
| 4 | 45 | 20.600 | 7.134 | 0.325 | 0.900 |  | 15.500 | 1.350 | 0.086 | -4.700 |
| 5 | 50 | 21.300 | 6.849 | 0.308 | 0.810 |  | 15.900 | 1.933 | 0.119 | -1.750 |
| 6 | 88 | 20.900 | 6.960 | 0.322 | 0.750 |  | 16.200 | 2.067 | 0.125 | -0.010 |
| 7 | 79 | 21.400 | 8.312 | 0.359 | 0.630 |  | 16.900 | 2.221 | 0.129 | -1.580 |
| 8 | 98 | 24.150 | 9.801 | 0.383 | 0.010 |  | 17.350 | 3.722 | 0.201 | -0.580 |
| 9 | 105 | 29.100 | 10.572 | 0.359 | 0.970 |  | 19.700 | 4.480 | 0.221 | -0.120 |
| 10 | 102 | 32.400 | 9.059 | 0.293 | 1.760 |  | 20.750 | 3.761 | 0.181 | 0.050 |
| 11 | 111 | 31.400 | 10.180 | 0.334 | 1.240 |  | 20.400 | 4.707 | 0.218 | -0.470 |
| 12 | 113 | 29.000 | 11.997 | 0.401 | 0.800 |  | 21.400 | 5.312 | 0.240 | 0.060 |
| 13 | 92 | 25.950 | 10.796 | 0.404 | 0.700 |  | 21.750 | 4.077 | 0.182 | -0.550 |
| 14 | 82 | 19.450 | 10.672 | 0.467 | 0.350 |  | 20.600 | 4.351 | 0.198 | -0.280 |
| 15 | 60 | 22.150 | 10.053 | 0.397 | 0.860 |  | 23.700 | 4.717 | 0.194 | -0.080 |
| 16 | 38 | 23.300 | 9.600 | 0.387 | 0.930 |  | 23.600 | 7.013 | 0.278 | -0.660 |
| 17 | 25 | 26.200 | 12.534 | 0.435 | 0.100 |  | 25.200 | 7.519 | 0.271 | -0.59 |
